# Healthcare professional and manager perceptions on drivers, benefits, and challenges of telemedicine: results from a cross-sectional survey in the Italian NHS

**DOI:** 10.1186/s12913-023-10100-x

**Published:** 2023-10-18

**Authors:** Grazia Antonacci, Elisabetta Benevento, Sveva Bonavitacola, Lorella Cannavacciuolo, Emanuela Foglia, Giulia Fusi, Elisabetta Garagiola, Cristina Ponsiglione, Alessandro Stefanini

**Affiliations:** 1 Department of Primary Care and Public Health, Imperial College London, National Institute of Health Research (NIHR) Applied Research Collaboration (ARC) Northwest London, London, UK; 2 Business School, Centre for Health Economics and Policy Innovation (CHEPI), Imperial College London, London, UK; 3 Department of Energy, Systems, Territory and Construction Engineering, University of Pisa, Pisa, Italy; 4 LIUC- Cattaneo University, Castellanza, VA Italy; 5 Department of Industrial Engineering, University of Naples Federico II, Naples, Italy; 6 Healthcare Datascience LAB, LIUC- Carlo Cattaneo University, Castellanza, VA Italy; 7 School of Economics and Business, Kaunas University of Technology, Kaunas, Lithuania

**Keywords:** Telemedicine, Telehealth, Benefits, Challenges, Drivers, Covid-19

## Abstract

**Background:**

The Covid-19 pandemic provided new challenges and opportunities for patients and healthcare providers while accelerating the trend of digital healthcare transformation. This study explores the perspectives of healthcare professionals and managers on (i) drivers to the implementation of telemedicine services and (ii) perceived benefits and challenges related to the use of telemedicine across the Italian National Health Service.

**Methods:**

An online cross-sectional survey was distributed to professionals working within 308 healthcare organisations in different Italian regions. Quantitative and qualitative data were collected through a self-administered questionnaire (June-September 2021). Responses were analysed using summary statistics and thematic analysis.

**Results:**

Key factors driving the adoption of telemedicine have been grouped into (i) organisational drivers (reduce the virus spread-80%; enhance care quality and efficiency-61%), (ii) technological drivers (ease of use-82%; efficacy and reliability-64%; compliance with data governance regulations-64%) and (iii) regulatory drivers (regulations’ semplification-84%). Nearly all respondents perceive telemedicine as useful in improving patient care (96%). The main benefits reported by respondents are shorter waiting lists, reduced Emergency Department attendance, decreased patient and clinician travel, and more frequent patient-doctor interactions. However, only 7% of respondents believe that telemedicine services are more effective than traditional care and 66% of the healthcare professionals believe that telemedicine can’t completely substitute in-person visits due to challenges with physical examination and patient-doctor relationships. Other reported challenges include poor quality and interoperability of telemedicine platforms and scarce integration of telemedicine with traditional care services. Moreover, healthcare professionals believe that some groups of patients experience difficulties in accessing and using the technologies due to socio-cultural factors, technological and linguistic challenges and the absence of caregivers.

**Conclusions:**

Respondents believe that telemedicine can be useful to complement and augment traditional care. However, many challenges still need to be overcome to fully consider telemedicine a standard of care. Strategies that could help address these challenges include additional regulations on data governance and reimbursements, evidence-based guidelines for the use of telemedicine, greater integration of tools and processes, patient-centred training for clinicians, patient-facing material to assist patients in navigating virtual sessions, different language options, and greater involvement of caregivers in the care process.

**Supplementary Information:**

The online version contains supplementary material available at 10.1186/s12913-023-10100-x.

## Introduction

The coronavirus disease 2019 (Covid-19) dramatically changed the ways of seeking and supplying medical care. During the pandemic, healthcare systems and individual healthcare providers implemented a variety of public health approaches to face the emergencies related to the spread of the Covid-19 virus, such as evidence-based contextual policies, intrahospital management strategies, community healthcare facilities, non-pharmaceutical interventions, improved surveillance, workplace prevention strategies, mental health interventions, and communication plans [1]. Among those strategies, the use of telemedicine tools rapidly increased across healthcare systems worldwide to respond to a combination of social distancing measures and the inability of primary and secondary care centres to receive non-Covid-19 patients [2–5].

The National Academy of Medicine defines telemedicine as “The use of electronic information and communications technologies to provide and support health care when distance separates the participants” [6], while the World Health Organization (WHO) has adopted a broader definition of telemedicine as “The delivery of health care services, where distance is a critical factor, by all health care professionals using information and communication technologies for the exchange of valid information for diagnosis, treatment and prevention of disease and injuries, research and evaluation, and for the continuing education of health care providers, all in the interests of advancing the health of individuals and their communities” [7, 8].

Telemedicine is particularly useful in circumstances where provider-patient interactions are not needed to occur in person, such as routine medical check-ups and mental health consultations [9, 10]. Reported benefits of telemedicine include increased access to care, shorter wait times, improved clinical results, high patient and clinician satisfaction and overall cost savings [11–14]. While, before the pandemic, telemedicine was primarily used to bring access to healthcare in remote areas with limited access to health services [9], the rapid implementation of telemedicine during the Covid-19 outbreak has been crucial to uphold the delivery of healthcare at a time when social distancing measures have been at the heart of fighting the contagion. In this context, telemedicine services allowed to free up space and capacity in acute hospitals and reduce the risk of infection transmission while ensuring continuity of care also for non-Covid19 patients [4, 15–18].

But the Covid-19 pandemic has resulted in the adoption of digital health technologies and changes to the way services are delivered at an incredible pace [5, 19–22]. While this is undoubtedly a great achievement demonstrating the capacity of healthcare systems to adapt quickly and to effectively transform the way they organise and deliver services when necessary, it is essential that this progress is maintained with sufficient resources in terms of finances, infrastructure and the workforce [2, 23–27].

When changes happen so rapidly and under exceptional circumstances, it is important to take a step back and question possible risks and downsides as well as the long-term impact and sustainability of these changes.

Challenges related to the implementation and use of telemedicine have been previously described by literature. They include greater medicolegal exposure, decreased continuity of care, reduced ability to perform physical examinations, workflow issues, clinician burnout, productivity and administrative burdens, and diminished personal connection with patients [26, 28–35]. A systematic review conducted by Scott Kruse et al. [36] identify several barriers associated with telemedicine implementation, including organisational (e.g. cost, reimbursement, legal liabilities), patient (e.g. age, education level, digital literacy) and staff, and programme barriers (e.g. complexity of technology for staff, resistance to change, licensing issues). A commonly reported barrier for patients is the inequality in access to telemedicine. For instance, older individuals are often less tech-savvy and comfortable with technologies, which may result in their inability to engage with telemedicine [37]. Low-income communities also demonstrate a lower uptake of telemedicine associated with lower educational levels and cultural or political factors [38]. As a result, on an international scale, lower- and middle-income countries, as well as certain higher-income countries, experience a digital divide, which refers to the gap between those who have access to technology, digital literacy and more specifically digital health literacy, and those who do not [28, 29, 39–44].

The challenges mentioned above are seen in all countries that use and aim to implement telemedicine [45] and are at risk of being amplified or simply overlooked in the case of a rapid implementation accompanied by profound changes in the regulatory, social, economic, and technological landscape [46].

During the pandemic, national bodies, healthcare providers, single healthcare professionals, and technology providers have undertaken many actions to respond innovatively and rapidly to continue providing services to patients using telemedicine. Some of these decisions might have been taken relatively short-term, and we don’t know how long they will be in place. For example, some technology providers provided free services during the pandemic, and healthcare providers may have understandably bypassed or accelerated staff training and change management support [47]. At the same time, national bodies significantly increased funding for telemedicine projects, accelerated procurement processes, and relaxed rules around information governance [47]. The Covid-19 pandemic has changed, in some cases temporarily, many countries’ regulations concerning telemedicine use [48]. A global increase in all forms of telemedicine for a wide range of speciality areas has been recorded, aided by quick governmental responses such as relaxation of compliance regulations [49] and, in some cases, the introduction of telemedicine legislation, such as South Korea, which has temporarily permitted its use [50]. Reimbursement was also made available where it was not the case before in certain countries [49, 51]. The United Kingdom embraced telemedicine at an unprecedented scale and has deployed a new digital-first approach to manage the streaming of care to the appropriate services, facilitated by relaxed General Data Protection Regulations (GDPR) [52]. Amid the pandemic (December 2020) the different Italian regional governments all agreed to define specific legal arrangements to integrate telemedicine into the national healthcare system, signing the document “National guidelines for the provision of telemedicine services”, which was licenced by the Ministry of Health [53]. Since then, the Italian regions have reacted in various ways and with different timescales.

These changes in the regulatory landscape have been accompanied by technological advancements, a greater offer of telemedicine solutions and a profound shift in people’s attitudes towards online interactions [54]. As a result, many activities that were previously conducted face-to-face are now conducted remotely, and people’s skills and confidence in using remote communication tools are significantly improved [55].

More research is required now to understand implementation drivers and challenges to the use of telemedicine following the dramatic changes at different system levels driven by the Covid-19 crisis [15].

As the use of telemedicine has increased exponentially since the Covid-19 pandemic onset, clinicians have had to quickly adapt to new ways of working [56, 57]. This involved learning new workflows and using new technologies to provide a substantial amount of virtual care. Sustaining and spreading the progress made during the pandemic will require a thorough examination of telemedicine’s impact in terms of systems and processes, clinical practice, quality of care, experiences of patients and staff, efficiency, and finances.

While we go back to a ‘new’ normal or the possible future waves, we must find ways to consolidate learning. More work is now required to learn from the experience and ensure that progress made during the pandemic will be effectively implemented, sustained, and spread in the future.

A large body of literature, including empirical studies, systematic reviews, and opinion pieces, describing benefits, challenges and success factors related to the use of telemedicine during the pandemic, has been increasingly produced [15]. However, knowledge about front-line staff perceptions is still limited [34, 58].

Rapid telemedicine adoption during the pandemic has raised many challenges for healthcare providers. Therefore, capturing the clinician’s perspective is critical to improving patient and clinician telemedicine experience and ensuring effective and sustainable implementation of these services [59].

Healthcare professional perceptions have been analysed in surveys [60–62] or other qualitative studies [58, 63–65]. Although these studies provide valuable insights into clinician perspectives on the use of telemedicine, they are limited to a single speciality [60, 61] and/ or local setting [62–65]. Furthermore, these studies mainly assess perceptions of benefits and challenges related to the use of telemedicine. Implementation drivers are less researched and primarily focus on factors related to the micro-system (e.g. team, patient-provider relationship, technological issues) and less on broader implantation drivers, such as organisational and regulatory aspects.

Perspectives of managers working with different roles within healthcare provider organisations could be useful to explore those factors. To our knowledge, no study has assessed the perspectives of healthcare professionals and managers covering a broad range of telemedicine services over different medical specialities at a national level. As telemedicine is likely to become an integral component of patient care even beyond the pandemic, it is crucial to understand clinician and manager perspectives about factors hindering and facilitating its implementation as well as its impact on patient experience, workflows, and the wider organisation [63].

This study aims to explore the perspectives of healthcare professionals and managers on: (i) drivers to the implementation of telemedicine services and (ii) perceived benefits and challenges related to the use of telemedicine across the Italian National Health Service (NHS).

The findings from this study will provide useful insights for healthcare providers and policymakers to improve telemedicine implementation across a wide range of medical specialities. This will allow embedding the positive work done during the pandemic while identifying and addressing the underlying challenges that health systems, organisations and individuals faced and are likely to face regarding telemedicine in the future.

## Methods

A cross-sectional survey was conducted in collaboration with the Italian Association of Management Engineers in Healthcare (IN.GE.SAN.) [66].

The study was designed following the approaches proposed by Forza [67] and Karlsson [68].

### Study setting

The study was conducted in the Italian NHS. In Italy, the 20 regional governments (19 Regions and 2 Autonomous Provinces) are responsible for planning and overseeing the management of healthcare services based on population needs. Regional governments designate local healthcare organisations’ boards and executive management, coordinate their action, supervise the achievement of results, and intervene in malpractice cases. At the local level, the NHS consists of Local Health Authorities, Hospital Enterprises (i.e., major hospitals with financial and technical autonomy [69]), National Institutes for Scientific Research (IRCCS), nursing homes and other public and private providers (e.g. laboratories).

The inclusion criteria of the study population were:

- Organization type: Local Health Authorities, public Hospital Enterprises, IRCCS, nursing homes, and other public and private healthcare providers.
- Country: Italy.
- Participant role: Healthcare professionals and administrative staff.

Purposive sampling was used to maximise the national territory’s coverage by reflecting the population distribution across the different Italian Regions. For pragmatic reasons, we utilised the IN.GE.SAN. contact database. To achieve the desired distribution of the sample across the various Italian Regions, we supplemented email addresses from the IN.GE.SAN. contact list with those obtained from the websites of healthcare organisations. This resulted in a total sample of 615 healthcare professionals and managers (Table 1).

### The survey instrument

The survey was designed through an iterative process. A comprehensive search of the literature was conducted to identify the main benefits, challenges, and implementation drivers of telemedicine [70–73] as well as innovation adoption and implementation frameworks [2, 3, 5, 74]. Empirical studies, opinion pieces, and evidence synthesis were included in the analysis, with a particular focus on studies describing the use of telemedicine during the Covid-19 pandemic. Literature findings and available telemedicine surveys (e.g., [54], [61]) were exploited for progressively devising the questionnaire. The questions were designed to obtain two complementary perspectives on the use of telemedicine: drivers and benefits/challenges. The final version of the survey includes nine items and is divided into two sections. Section 1 asked respondents to provide general information on their organisation, key characteristics of the implemented telemedicine services, and the drivers that have guided telemedicine implementation. Section 2 assessed healthcare professionals’ and managers’ perceptions of the benefits and challenges of telemedicine use. Furthermore, healthcare professionals were asked to express their opinion on the usefulness and ease of use of telemedicine from the patient’s point of view. The survey includes closed-ended questions where respondents need to indicate their level of agreement on a 5- point Likert scale (e.g., strongly agree, agree, neither agree nor disagree, disagree, strongly disagree) as well as multiple-choice and open-ended questions. The questionnaire is included in the Supplementary Materials 1 reporting for each item the related references (when appropriate).

To validate the questionnaire, the research team involved a group of health managers and health professionals, with experience in telemedicine service implementation, to assess the content validity of the entire questionnaire [75–77]. The items that did not achieve an adequate level of content validity were adjusted until they were deemed satisfactory or were removed. Then a pilot testing of the questionnaire was conducted. The survey was administered to five healthcare professionals and managers to examine the clarity of the questions, to evaluate the measurement validity, and to check the administration procedures [68]. This pre-test was carried out before the survey was frozen and distributed to the study population.

The final version of the questionnaire was implemented through an online questionnaire platform (Google Forms) thus allowing the remote completion of the questionnaire and the automatic collection of answers. To be noted that Google Forms also permitted each user to fill in the questionnaire once since it checks the email account before the start of the questionnaire.

### Data collection

Data were collected for 4 months between June and September 2021. An email with a description of the project’s scope and a link to the questionnaire was sent out by the research team to 615 healthcare professionals and managers on 8th June 2021. Contact details of the research team were provided in case the respondents had any questions. The survey was estimated to take approximately 15 min to complete. To increase the response rate, respondents were followed up twice (on 25th June 2021 and on 2nd September 2021) via a reminder email inviting them to visit the survey homepage and complete the survey. Data were extracted on 27th September 2021. For all participants, the survey included mandatory and not mandatory fields. In addition, the manager perspective was required to complement the healthcare professional perspective only for relevant sections.

### Data analysis

Data were cleaned before starting the analysis, which was conducted using Microsoft Excel 2019. Descriptive statistical analysis and different forms of representation have been used according to the typology of questions.

Concerning the Sect. 1, the descriptive statistical analysis gives information on the interviewed sample and the organisations where they work.

Concerning the Sect. 2, the questions may be open-ended or closed questions. The analysis of the open-ended questions has required a thematic analysis of the answers to group them in clusters. Then, a descriptive statistical analysis has been performed on the emerged clusters.

The closed questions concern two typologies: five points Likert scale and preference order.

The analysis has been performed coherently with other empirical analysis using the same typologies of closed questions [78–81]. The analysis of closed items related to Likert scale included a first step of calculation of the per cent distribution for each point of the scale. This per cent distribution was presented in three categories: (i) *4 + 5* - strongly agree and agree, (ii) *3* - neither agree nor disagree and (iii) *1 + 2* - disagree and strongly disagree [81]. In this way, it is possible to immediately catch a positive, negative, or neutral inclination related to a specific item. Instead, the analysis of closed items related to a preference order involves the count of the preferences given for a specific item and the calculation of the relative percentage.

Perspectives of healthcare professionals and managers were first analysed separately also according to the typology of organization where they work. The difference in views and organizations was then assessed and only reported when there was a significant mismatch.

### Ethics and consent

According to Italian legislation, this project did not require formal ethical approval because it collected general opinions that do not include clinical data and neither personal data. Ethics approvals in Italy are regulated by the Regulation of the Ethics Committee of the Higher Institute of Health (Istituto Superiore di Sanità, Rome 12th May 2015). This Regulation stipulates that projects with epidemiological, medico-social, and evaluative contents need evaluation, approval and monitoring of trial protocols only if they contain personal data according to the Italian legislative decrees on clinical trials and function of the ethics committees (decreto legislativo 24 giugno 2003, n.211, “attuazione della Direttiva 2001/20/CE”; decreto ministeriale 8 febbraio 2013). The official definition of “personal data” is specified by the National Data Protection Authority (Garante per la Protezione dei Dati Personali, https://www.garanteprivacy.it/home/diritti/cosa-intendiamo-per-dati-personali– Regolamento (UE) 2016/679 art.9). According to this Regulation, the term “personal data” applies to data containing information about first and last name, images, tax code, IP address and license plate number. None of this information was collected in this study. The platform on which the anonymous questionnaire was completed does not allow us to trace the IP address of the person who connected to the survey. The data collected were absolutely anonymous, and tracing the identity of the survey participants was not possible. Moreover, this study did not involve patients and clinical data. Informed consent was obtained from all subjects and/or their legal guardian(s). Consent information was included in the invitation and reminder emails. Participation was entirely voluntary, and consent was implied by responding to the survey. Data were anonymised and treated with the utmost confidentiality in accordance with applicable Italian data protection laws.

## Results

A total of 124 participants completed the survey, resulting in about 20% (124/615) response rate (Table 1). Two-thirds of the respondents were healthcare professionals (86/124), while one-third were managers (38/124). This reflects the distribution of these two groups within healthcare providers in Italy. The respondents were balanced in terms of sex (46% women and 54% men), age distribution (average 48 years), and years of service (average 21 years of service). Most respondents belong to public health organisations (90%), as the health service is primarily public in Italy. The health organisations of the respondents were located throughout the entire country, with 14/20 Regions involved, covering more than 90% of the Italian population (Table 1). The regional distribution of respondents is consistent with existing literature, reports, and recent data from the Italian Ministry of Health, highlighting disparities in the adoption of telemedicine services across Italy [82–85]. Notably, there has been a significantly higher uptake in Northern Italy compared to Southern Italy.

**Table 1:** Distribution of the number of surveys sent, number of respondents and response rate by Region (three respondents didn’t specify the Region)

Region	Number of surveys sent	Number of respondents	Response rate
Lombardia	104	31	30%
Lazio	60	8	13%
Campania	59	2	3%
Veneto	51	37	73%
Sicilia	50	5	10%
Emilia-Romagna	46	7	15%
Piemonte	44	13	30%
Puglia	41	4	10%
Toscana	38	5	13%
Calabria	19	0	0%
Sardegna	17	2	12%
Liguria	16	0	0%
Marche	15	0	0%
Abruzzo	13	0	0%
Friuli-Venezia Giulia	12	1	8%
Trentino Alto Adige	11	4	36%
Umbria	9	1	11%
Basilicata	6	1	17%
Molise	3	0	0%
Valle d’Aosta	1	0	0%
(not specified)		3	
**Total**	**615**	**124**	**20%**

### Drivers to the implementation of telemedicine services

**Fig. 1:**
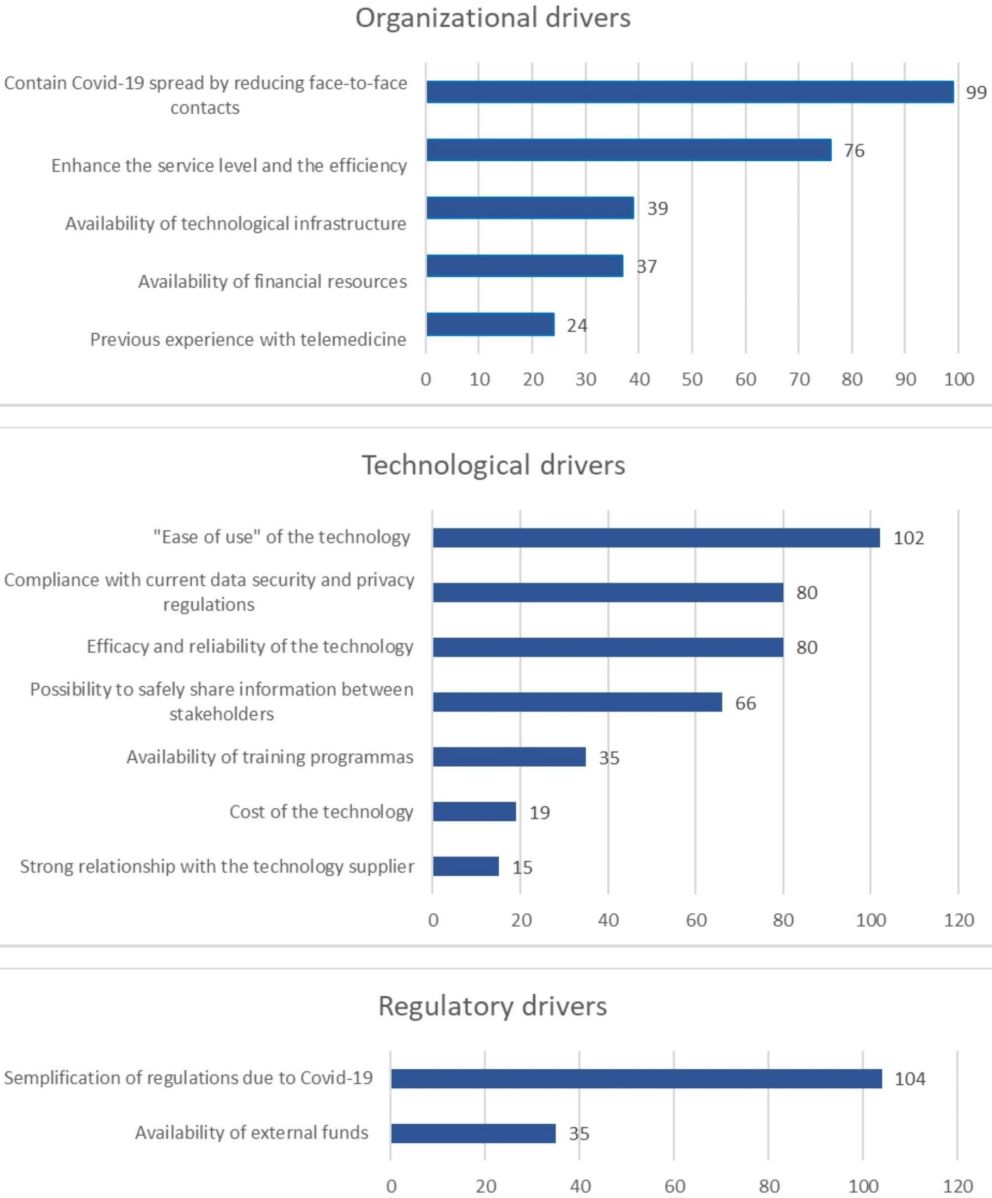
Drivers related to the adoption of telemedicine service during the Covid-19 period

The main factors driving the adoption of telemedicine services during the Covid-19 pandemic have been grouped into three macro-areas: (i) organisational, (ii) technological, and (iii) regulatory (Fig. 1). The answers are aligned among clinicians and managers.

*(i) Organisational drivers*.

The most cited driver to the implementation of telemedicine is the attempt to contain the spread of the Covid-19 virus by reducing face-to-face contacts (80%, 99/124). This is followed by opportunities to enhance the quality and efficiency of care (61%, 76/124). Previous experience with telemedicine (19%, 24/124) and the availability of technological infrastructure (31%, 39/124) and financial resources (30%, 37/124) are instead perceived by fewer respondents as relevant drivers to telemedicine adoption during the pandemic crisis.

*(ii) Technological drivers*.

The most commonly cited driver related to the technology is relative to its “ease of use” for healthcare professionals, patients, and caregivers (82%, 102/124). This is followed by the efficacy and reliability of the technology (64%, 80/124) and compliance with current data security and privacy regulations (64%, 80/124). The possibility to safely share information between healthcare providers and patients, as well as across different organisations, was also perceived as a key driver to the adoption of telemedicine by more than half of respondents (53%, 66/124). Drivers that respondents have less cited include a strong relationship with the technology supplier (12%, 15/124), the cost of the technology (15%, 19/124), and the activation of training courses (28%, 35/124).

*(iii) Regulatory drivers*.

The simplification of regulations due to the Covid-19 outbreak – in terms of financial accounting, privacy rules, technological purchasing, and governance – was the most cited driver of the adoption of telemedicine services (84%, 104/124). Moreover, similarly to what emerged in the organisational drivers, the availability of external funds (e.g., national funds) for acquiring the technology is not perceived as a key driver to the activation of telemedicine services (28%, 35/124).

### Perceived benefits and challenges related to the use of telemedicine

Both healthcare professionals and managers perceive telemedicine as a valuable service to improve patient care and believe that the telemedicine solutions introduced during the pandemic can continue to be used beyond the Covid-19 emergency (96%, 119/124). However, 66% (57/86) of healthcare professionals believe that telemedicine can’t completely substitute face-to-face care due to challenges related to the patient-doctor relationship, the need for a physical examination to diagnose and treat some health conditions and the day-to-day organisation of clinical work.

89% (110/124) of respondents shared their perspectives on the expected benefits of telemedicine through an open-ended question. These are mainly related to the positive impact on healthcare provider operations, patient travel, and direct patient care. The reduction of the waiting list for inpatient and outpatient appointments was indicated by respondents as the main benefit of telemedicine, followed by the decrease in Emergency Department (ED) attendance and the reduction of time and cost for patient and clinician travel. Other benefits perceived as relevant by respondents include the possibility for patients to be continuously and timely monitored by healthcare professionals and to get in touch with them more often, increasing the efficiency of follow-ups. Another benefit perceived as important by respondents is the possibility of ensuring a better outreach service, as telemedicine allows greater coverage of health services in areas that are generally difficult to reach (e.g., mountainous areas). Three healthcare professionals also highlighted how telemedicine could facilitate secondary care integration with other care settings.

**Fig. 2:**
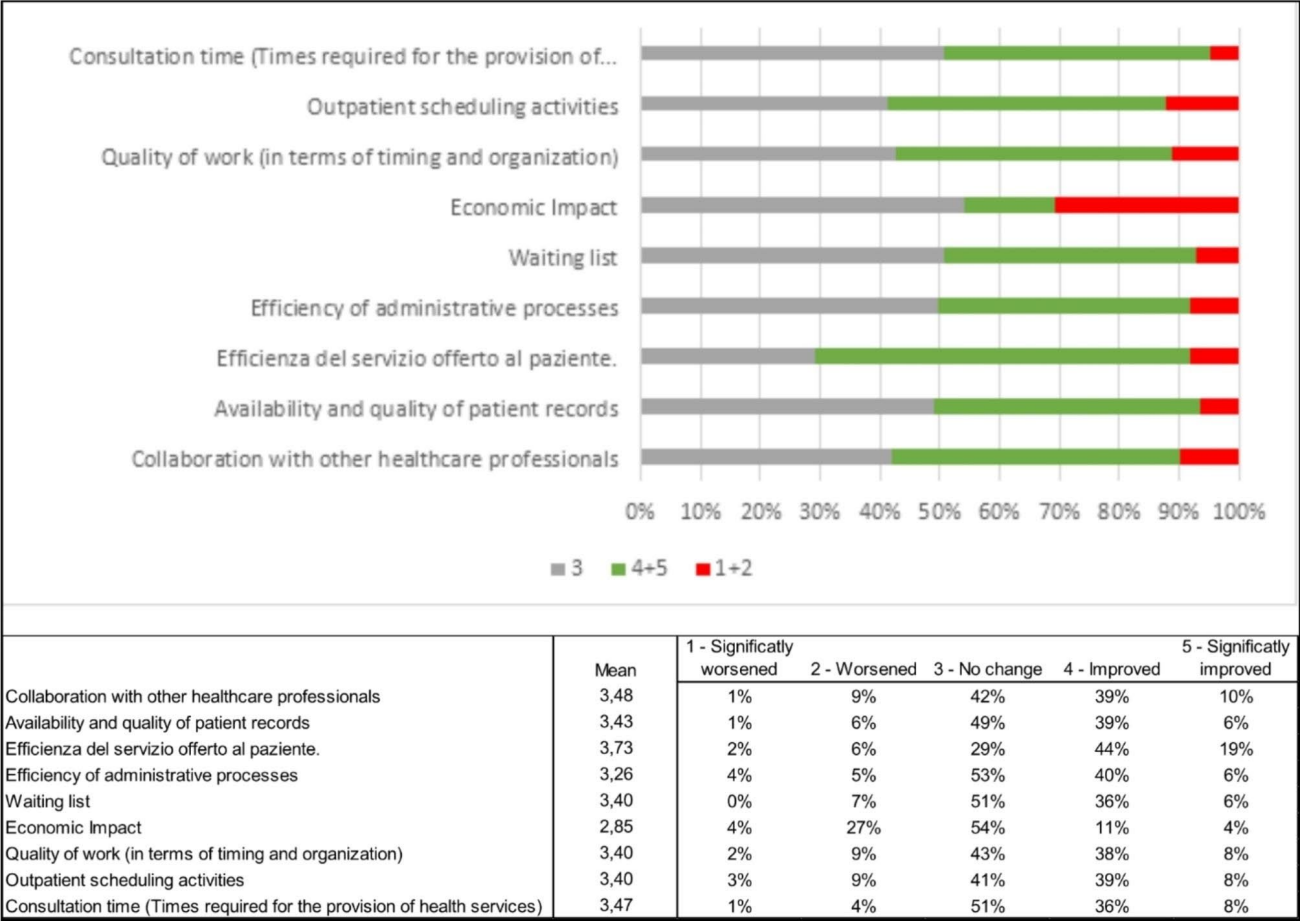
Impact of telemedicine on operational and organizational aspects

By looking more closely at the perceptions about the impact of telemedicine on operational and organisational aspects, data reveal that both healthcare professionals and managers believe telemedicine can positively impact these areas (Fig. 2).

Perceptions of telemedicine’s impact on operational aspects were further explored through an optional question asking healthcare professionals whether the adoption of telemedicine impacted their day-to-day work activities. 77% (66/86) of the healthcare professionals answered this question, and 38% (25/86) of them filled in an open-ended question asking to explain why. 48% (32/66) of respondents perceive that the work routine improved, and 40% (26/66) believe it didn’t change. Only 12% (8/66) of respondents believe that the work routine worsened as a result of the introduction of telemedicine, specifying that it was mainly due to the lack of IT platforms specific for telemedicine services and to the poor integration between telemedicine and traditional care processes leading to duplication of activities and staff burden.

Healthcare professionals were also asked through an optional question if they believe that telemedicine services are more effective than traditional care in delivering better patient outcomes. Data reveal that despite clinicians acknowledging the beneficial role of telemedicine in enhancing the frequency of doctor-patient interactions, only 7% (5/67) of respondents believe that telemedicine services are more effective than traditional care. In comparison, 48% (32/67) think that telemedicine is less effective, and the remaining 40% (30/67) believe that they are equally effective.

**Fig. 3:**
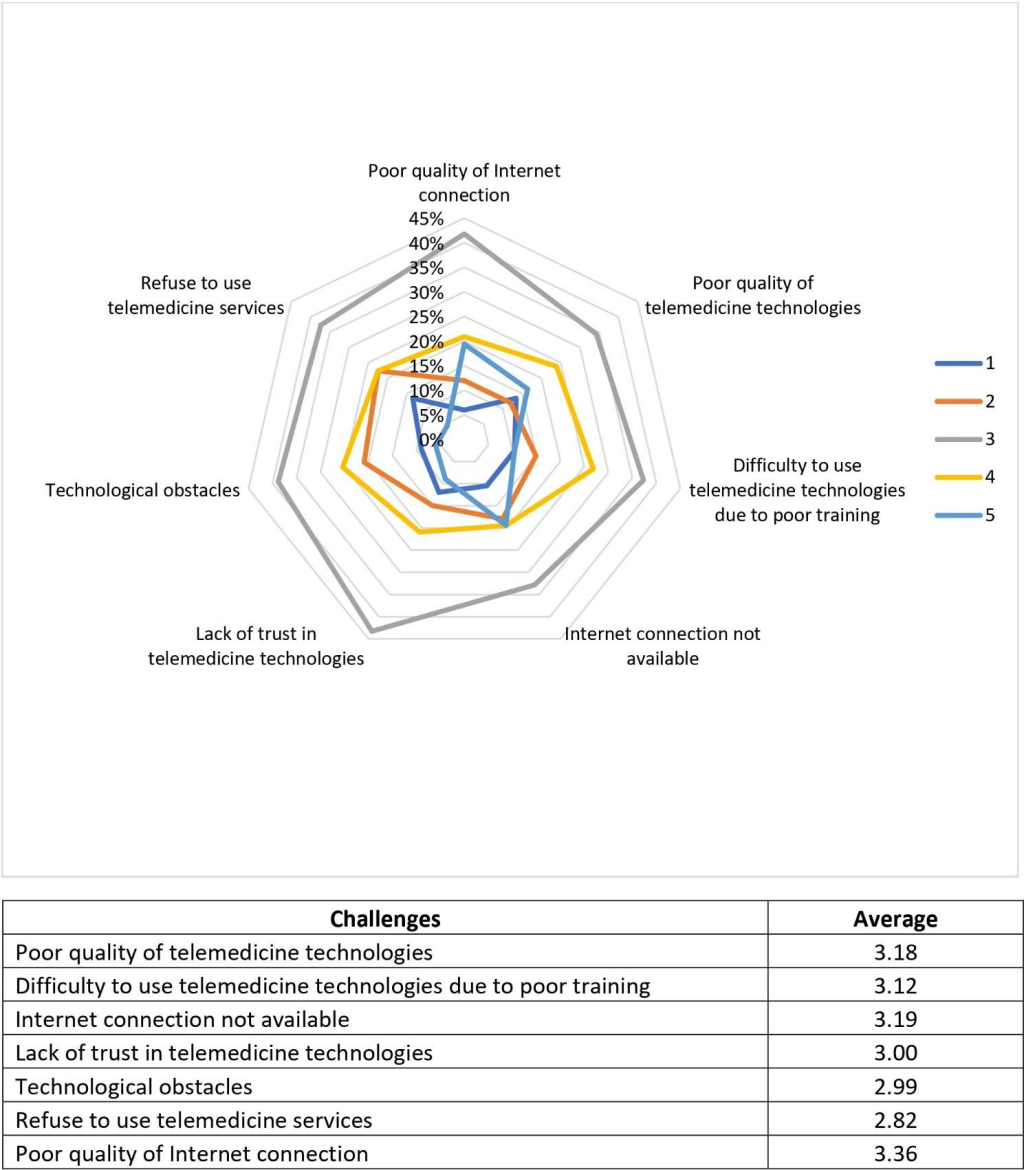
Challenges with the use of telemedicine for healthcare professionals

Moreover, healthcare professionals’ barriers to the use of telemedicine were investigated through closed-ended questions revealing that common challenges are related to the low quality of internet connection and other technologies, the lack of trust in the technology used, and the difficulty in using the technology (Fig. 3).

**Fig. 4:**
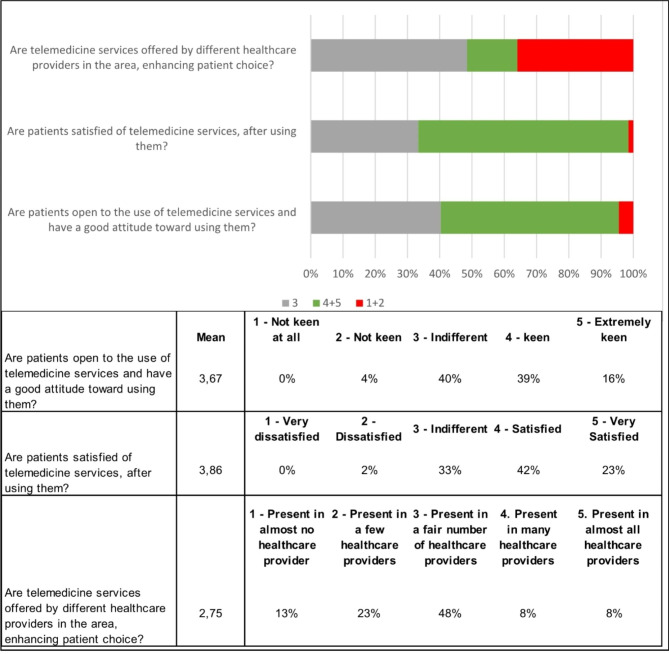
Patient acceptability and satisfaction

Looking at the patient side, healthcare professionals believe that patients have a positive attitude toward telemedicine, both in terms of acceptability and user experience (Fig. 4).

**Fig. 5:**
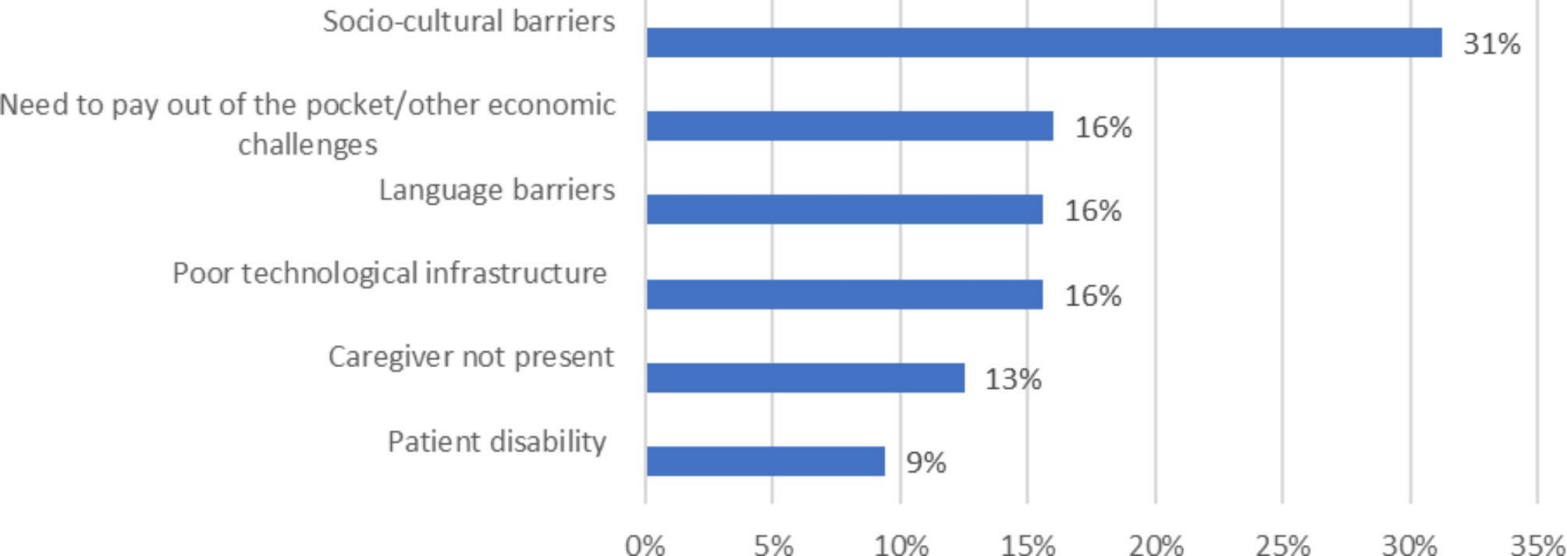
Barriers to the use of telemedicine for specific groups of patients

However, some challenges are still related to the equity of access and technology usage. Healthcare professional concerns are associated with the poor offer of these services in some territories and specific barriers some patient groups face (Fig. 5).

Healthcare professionals (64%, 43/67) perceive that the most relevant barriers to the use of telemedicine for patients are related to socio-cultural factors, followed by technological and linguistic challenges and the absence of caregivers. 75% (50/67) of respondents believe that the presence of a caregiver is essential for the use of telemedicine services to assist elderly patients and people with disabilities.

## Discussions

The Covid-19 pandemic presented new challenges and opportunities for patients, their families, and healthcare professionals while also speeding up the current process of digital transformation in healthcare [86]. As telemedicine will increasingly become an integral component of healthcare services provision, it’s important to understand front-line staff perception and experience with its use. In this paper, the perceptions of healthcare professionals and managers on benefits, challenges, and drivers to the implementation of telemedicine services have been explored across the Italian NHS during the pandemic period.

We found that both healthcare professionals and managers believe that using telemedicine services can positively impact patient care and operational and organisational aspects. This is in line with other studies exploring clinician perspectives on telemedicine during the Covid-19 crisis [34, 58–61].

### Drivers to the implementation of telemedicine services

As expected, study participants agreed that the rapid adoption of telemedicine services throughout the Covid-19 pandemic was mainly due to the need to respond to the emergency by reducing face-to-face contact [4, 64, 65, 87]. Italy was one of the first European countries to be severely impacted by COVID-19, with its healthcare system overwhelmed by the influx of patients [88]. In this context, telemedicine was critical in reducing face-to-face contact and limiting virus propagation while reducing the burden on hospitals and ensuring continuity of care [89–91].

Findings also confirm previous studies showing how regulatory aspects are a barrier to the adoption of telemedicine and how easing these barriers can facilitate the implementation of these services [92–99]. In Italy, like in other countries, the use of telemedicine has remained relatively limited in recent years due to several regulatory obstacles, such as poor reimbursement regulations, complex technology purchasing processes, and a lack of data governance and privacy guidelines [61, 100]. Italy features a decentralised healthcare system in which regions manage their own healthcare services. As a result, legislation has become fragmented, with different regions implementing telemedicine regulations and reimbursement schemes to varying degrees [101]. The lack of a unified regulatory framework presents difficulties for healthcare providers and businesses seeking to operate on a national scale. Because telemedicine involves the transmission of sensitive patient data, data security and privacy are critical concerns. Telemedicine services in Italy, like those in other European nations, must comply with the European Union’s General Data Privacy Regulation (GDPR) and national data privacy legislation. Compliance with these standards can be difficult to achieve, especially for smaller healthcare providers and startups with limited resources [102]. Remote contact-specific data privacy regulations were absent in many countries, including Italy, before the epidemic and then implemented [61, 103]. Similar to this, particular data protection regulations for telemedicine that were not previously available in most situations became available during the pandemic [61, 94–98, 104–107]. Moreover, before Covid-19, most regional healthcare systems and insurance providers didn’t pay for telemedicine consultations [61]. Telemedicine reimbursement schemes in Italy vary across regions, with certain regions covering specific telemedicine services while others do not. The inconsistency of reimbursement can be a hurdle for healthcare providers looking to implement telemedicine services [108]. To encourage the use of telemedicine during the pandemic, some regions in Italy expanded their reimbursement policies to cover telemedicine services [108]. This enabled healthcare providers to be compensated for remote consultations, making telemedicine more appealing to both providers and patients. The Italian government also implemented temporary adjustments to existing regulations to promote telemedicine during the COVID-19 pandemic. For example, the Ministry of Health established guidelines on the use of telemedicine during the crisis, offering a clearer structure for healthcare practitioners and organisations to follow [53, 109]. However, although in many countries changes in national and local regulations and guidelines during and beyond Covid-19 have introduced specific reimbursements for telemedicine services and improved data protection and privacy for telemedicine, concerns about these issues have been still reported in recent studies by physicians in Italy and in other countries as a major challenge to the adoption of telemedicine [59–61, 103]. During the pandemic, local, national, and international governments around the world also used financial incentives to encourage healthcare providers to adopt telemedicine services, such as grants for technology acquisition and implementation. The Italian government allocated funds to support the expansion of telemedicine services during the pandemic, by enabling healthcare organisations to invest in the infrastructure and equipment required to deliver remote consultations and monitor patients at home [110]. Several Italian regions have allocated funds to support the development and deployment of telemedicine solutions. For example, the Lombardy area, which was particularly heavily struck by the epidemic, allocated significant funds to develop telemedicine services and assist remote patient monitoring [111]. Italy also benefited from European Union (EU) financing to boost the digitalization of healthcare services, particularly telemedicine. The EU’s “Next Generation EU” recovery fund, established in response to the COVID-19 crisis, provided billions of euros to member states, including Italy, to assist digital transformation projects [112]. This is reflected in our findings revealing that, differently from previous literature [113], the availability of financial resources to acquire or implement telemedicine services was not regarded as a key driver of adoption during the pandemic.

### Perceived benefits related to the use of telemedicine

According to previous literature, respondents perceive telemedicine as useful in improving patient care even beyond the pandemic crisis [63, 114–116]. The possibility to enhance patient care, improve operational aspects and reduce travel for patients and healthcare professionals were perceived as key benefits of the use of telemedicine both during and beyond the pandemic period. Our findings corroborate those of other studies that found telemedicine to have positive effects on follow-up care efficiency, the ability to see patients more frequently when necessary, improved rapidity of care delivery (shorter wait times for appointments, shorter consultation times), and a reduction in missed appointments [15, 55, 58, 92, 105, 107, 117–119]. Through telemedicine, patients can avoid travelling to and from appointments, taking less time off work, or waiting in waiting rooms. Avoiding travel saves time and money on parking, transportation, childcare, or other carer costs. Patients who face physical or financial obstacles to access, such as the elderly, those with mobility issues, people who live in remote locations, and people with low incomes, stand to benefit especially from this [58, 92–95, 106, 107, 117–123].

Our findings also corroborate previous research describing how telemedicine can improve the quality of work of healthcare professionals as telemedicine gives doctors more flexibility with their schedules and patient availability [58]. Moreover, remote working allowed those who were more at risk of contracting the virus during the pandemic period to continue working (such as senior physicians).

In terms of healthcare infrastructure and resources, Italy has considerable regional disparities, with some rural and remote areas having limited access to healthcare facilities [124]. In line with previous literature, participants concurred that telemedicine could improve patient access to care, particularly for individuals who reside in rural, underdeveloped, or resource-constrained locations [55, 60, 92, 93, 104, 105, 107, 120, 123, 125].

Regarding improving operational and organisational aspects, findings confirm previous literature demonstrating telemedicine’s positive impact in reducing patient waiting lists and ED attendance [126–129]. Participants also believe that adopting telemedicine services could potentially lead to an overall improvement in administrative and care processes.

### Perceived challenges related to the use of telemedicine

Some participants expressed concerns about poor coordination of workflows leading to duplication of tasks and additional burdens for clinicians. According to earlier research, the burden of telemedicine in Italy like in other countries can be related to the challenges of integrating telemedicine into routine clinical practice, the complexity and lack of interoperability of platforms, poor data integration among devices, and the requirement for additional time for virtual consultations [15, 61, 92, 98, 99, 119, 123, 130–132]. Other studies described how telemedicine services led to the duplication of visits when the virtual assessment was insufficient [58], or to an increased workload for clinic administrative personnel to coordinate access to virtual treatment (including assisting patients and caregivers in choosing between phone and video conferences and facilitating technology troubleshooting) [64].

Moreover, most respondents agree that telemedicine services can’t completely substitute patient-clinician and clinician-clinician face-to-face interactions throughout the care process. Only a few respondents (7%) perceive telemedicine services as more effective in delivering better patient outcomes than traditional (face-to-face) care. Our findings corroborate previous literature reporting that this is mainly due to challenges with the patient-doctor relationship, cases in which physical examination is needed or recommended, and the organisation of clinical work [28, 29, 34, 41, 63, 132–135].

Difficulties related to the decreased ability to perform physical examinations have been widely reported in other studies exploring the healthcare professionals’ perspective on the use of.

Telemedicine [58, 60]. Clinicians believe that while there are some situations in which face-to-face consultations can be avoided, such as those in which a diagnosis can be made based mainly on the patient’s medical history (i.e., no physical examination is required), there are other circumstances in which a physical examination is necessary or advised [60, 136]. Recent papers describe the attempt to solve this issue by releasing instructions for efficient virtual examinations [137, 138]. However, in Italy, specific instructions to guide the use of telemedicine to diagnose and treat patients with specific medical conditions were not present at the time the survey was conducted. Physical examination is essential not only for efficient clinical practice - it is also an integral component of the doctor-patient therapeutic connection [58, 139]. Studies have also drawn attention to the possibility that telemedicine technologies could jeopardise the continuity of treatment and the therapeutic relationship, two features of care delivery crucial to clinical practice that have significant implications for patients and doctors [28–30, 58]. Effective treatment of mental, emotional, and behavioural health issues depends on the relationship between the patient and the clinician [30]. The Covid-19 pandemic has highlighted the importance of establishing social connections remotely for therapeutic human relationships in addition to assuring the safety and effectiveness of care delivery [29, 30]. In a virtual care context, maintaining continuity of care and establishing therapeutic relationships with patients necessitates learning new techniques for establishing deep connections through everyday interactions [30].

Our findings also show how these concerns and obstacles associated with technology-mediated communication between patient and doctor, as well as issues concerning poor quality and difficulty with technology use, can have a detrimental impact on clinician trust in telemedicine and its widespread adoption [63].

Moreover, according to earlier studies, a major obstacle to the adoption of telemedicine is related to technological limitations faced by both patients and healthcare professionals [58, 61, 92, 96, 99, 100, 107, 118, 131, 140]. This is especially true when using virtual tools to make diagnoses and the difficulty of learning new software while under time constraints at the beginning of the pandemic [58]. The impact of technological obstacles on both patients’ access to care and doctors’ ability to deliver high-quality care should not be underestimated. This is consistent with research findings showing that some patients and providers face technological literacy and logistical challenges when participating in telehealth visits, particularly given the variety of technologies available and/or the ways in which some medical practices have shifted technologies [28, 41, 135]. Previous studies show how patients’ concerns with telemedicine are related to a lack of knowledge and skills to use these technologies effectively, restricted access to the necessary equipment (e.g. cameras, email, smartphone), difficulties with installing applications and limited access to the internet [58, 60, 63]. In some parts of Italy, particularly in rural areas, limited internet access or low bandwidth can hamper the effective implementation of telemedicine services. A commonly reported remedy when a patient is unable to connect for a video appointment is switching to a phone conversation, as this was the most straightforward option for both doctors and patients [58, 64]. However, this is not a desirable option as, according to research, the lack of visual information limits clinicians’ capacity to evaluate the patient’s condition (literacy, language barrier, difficulty asking/responding to inquiries, etc.) and the patients’ “homes” actual surroundings, causing diagnostic difficulties [58, 63, 64].

Finally, our findings show that although healthcare professionals perceive a positive attitude of patients toward telemedicine, they believe several barriers need to be overcome to improve equity of access and use. In Italy, there is a considerable digital divide, with older people and people from low-income families frequently lacking access to digital devices and the essential digital literacy skills to fully benefit from telemedicine services [141–143]. Our results corroborate a large body of literature showing that problems with access and use of the technologies are exacerbated for specific groups of people, such as older adults, people with hearing impairment, disabilities, or other vulnerabilities (e.g., low-income) as well as for people not speaking the local language [92, 120, 122]. In line with our findings, these studies emphasise the critical role of caregivers in assisting patients with technological challenges, the description of the medical history and the development of a treatment plan, which is especially necessary when seeking virtual care [64].

### Implications

With this study, we contribute to a growing body of literature exploring the use of telemedicine from the front-line staff perspective during the Covid-19 pandemic. Because the increasing use of telemedicine during the pandemic drastically impacted how care services were provided, this study allowed us to gather staff perspectives in a different situation compared to earlier studies. We believe that our findings are generalisable since our survey included individuals from various Italian areas and application contexts in terms of medical specialisations and technologies.

The Covid-19 pandemic has significantly accelerated the adoption of telemedicine services in Italy [101]. Healthcare professional and manager perceptions investigated in this study suggest that, while telemedicine cannot completely replace face-to-face care, it can improve patient care, patient experience, and operational and organisational performance if used in conjunction with traditional care practices. As the use of telemedicine in Italy and worldwide grows in parallel with the continued use of face-to-face visits, it is critical to develop strategies to ensure that this mode of care delivery is, secure, and equitable in both routine and emergency scenarios.

This study reveals that regulatory changes occurring in Italy amid the Covid-19 pandemic were perceived as key drivers to the adoption of telemedicine services. Study participants pointed out how compliance with data governance and regulations is a critical factor for the adoption of telemedicine services and how the simplification of regulations (e.g. regarding financial accounting, privacy issues, technological purchasing) was a key driver to the adoption of telemedicine during the pandemic. Several regulations and guidelines have been implemented by central and local governments in Italy since the start of the pandemic [53, 109, 110]. However, numerous regulatory problems have been encountered in the attempt to regulate telemedicine, as have numerous efforts by governmental administrations to develop a robust and cohesive legal framework. The highly rapid progress of technology, as well as the ongoing evolution of European cybersecurity rules, make defining a suitably complete regulatory scenario difficult [144]. The applicable legal regulations for telemedicine are still insufficient, and the current ones are unclear; there are no consistent regulations at the European level for healthcare and the practise of medicine [145]. As a result, the use of this instrument in public services remains a challenge. Additional standards and regulations are required as the industry evolves, as some of those in place were only temporary during the Covid-19 outbreak [3]. A greater effort should be made by governments and regulatory bodies to enhance data protection and privacy and provide clearer rules and guidance on the reimbursement of telemedicine services. Moreover, technology access could be improved by retaining and upgrading measures established during the pandemic to ease purchase processes and by keeping supporting technology acquisition and implementation financially.

Findings from this study also highlight that attention should be paid to fully integrating telemedicine services into current care processes and systems. The availability of evidence-informed guidance would facilitate this integration by improving clinician trust in the use of the technology and would eventually lead to increased efficacy and efficiency of care by avoiding the repetition of visits for cases where virtual care was not the preferable care modality [34, 64, 146, 147]. So far little guidance is available in Italy to help clinicians, patients, and caregivers understand how to integrate virtual care safely and effectively into clinical practice. Further guidelines should be developed by national and international medical associations and scientific communities to indicate evidence-based practices for the use of telemedicine in different medical specialities. These guidelines should outline criteria to suggest cases where telemedicine is more appropriate and where instead, a physical examination is needed or recommended [128, 148]. Moreover, as the stress placed on healthcare systems by the emergency crisis relaxes, the development and implementation of operational guidelines within and across health and care organizations would also help to redesign and standardise workflows, as well as redefine roles to optimise the use of virtual care alongside traditional care services [34, 61, 65, 149–152]. Findings from this study also highlight how care and administrative process efficiency could be further improved by reducing the heterogeneity of the digital platforms and by making sure that tools used within each remote care process are interoperable. The problem of the heterogeneity of information systems and data integration in Italy is further compounded by the fragmentation of regional health systems. In 2022 an initiative has been launched by the central government to build a National Platform for Telemedicine, which should potentially guarantee the interoperability and integration across the different digital ecosystems [153]. Guidelines aimed at providing a single strategic direction at national level to improve data sharing and interoperability are also available, however their implementation across the national territory is still a significative challenge [154].

Inequalities in access to care and technology usage were also mentioned as a key concern to study participants’ use of telemedicine, as related to disparities in service offered across national territories, the presence of technological infrastructure, as well as economic, socio-cultural, technological, and linguistic challenges, and the absence of a caregiver. Guidance and regulations aimed at increasing the equity of access to care for specific groups of patients who might have considerable issues accessing and using telemedicine services could help to reduce these inequalities. National guidelines on this are relatively new in Italy, and they did not exist at the time the survey for this study was administered. In November 2022 the Italian Government published the “National guidelines for telemedicine services – functional requirements and service levels” aimed at improving equity of access and efficiency of telemedicine by outlining patient eligibility criteria for virtual care based on individual patient characteristics, such as clinical aspects, availability of the required technology, digital literacy, patient autonomy, or presence of a caregiver [155]. Although this represents a significant step forward, many challenges still need to be faced with implementing these guidelines in practice. Other approaches that could be implemented to reduce the potential disparities in care access as emerging from this study include: increasing investments to enhance the technological infrastructure and connectivity, expanding the availability of telemedicine services to enable access and increase patient choice in underserved areas, providing technologies at reasonable prices for people in need, offering different language options and involving caregivers as much as possible in the care process.

Poor digital literacy, poor training, and difficulty in using telemedicine technologies have also been reported by the study participants as key barriers to the effective use of telemedicine. More assistance to facilitate video visits for patients and professionals could help to overcome these barriers. In Italy digital health skills are still not fully embedded into clinicians’ academic training [156]. Clinical educators should focus on integrating new telemedicine competencies into learner curricula and practice. Telemedicine education for healthcare professionals should emphasise integrating learners into workflows and assisting patients in navigating virtual visits by incorporating patient-centred care principles [63]. This training should encompass the social and emotional components of care delivery to provide clinicians with guidance and skills for remotely nurturing and developing the patient-physician relationship, especially with new patients [58], as this has been reported by the study participants as a critical concern related to the use of telemedicine services. On the patient side, creating patient-facing materials to assist patients in preparing for and navigating virtual sessions (e.g. pre-visit information on camera/body placement, clothes, and setting) has also been suggested as an effective approach to improve the efficiency and quality of telemedicine visits [157, 158] and overcome some technical challenges emerging from this study. To close the digital divide, organisations must look deeper into their patients’ telehealth experiences and engage them in identifying the constraints that impede their capacity to participate in video sessions [64, 159]. In this study, we did not directly survey patients, and all identified benefits and challenges, as well as perceptions, are based solely on clinician experience. Future research could elicit these perceptions directly from patients to better understand their challenges and perceived benefits of telemedicine. A qualitative study could be conducted to explore patient telemedicine experiences and develop patient resources and interventions to improve access to technology and better screen for and encourage patient eHealth literacy [160].

Healthcare professional perspective could also be further explored in future studies. Previous research shows that clinical experience and burnout are improved by training and a high technical knowledge and experience level [63]. As clinicians get more comfortable with virtual visits and new clinical support is added, provider experiences with telemedicine should be reassessed.

Lastly, more research could be conducted to understand the economic implications of healthcare provider reimbursement for virtual care and technology and operational aspects related to widespread virtual care deployment in clinical practice.

### Limitations

A key limitation of this survey was the low response rate (20%). As reported in similar studies, experiencing this can be attributable to the burden that healthcare providers faced during the pandemic [60]. The low response rate might have resulted in selection bias of study participants, leading to an overestimation of positive attitudes toward telemedicine, as healthcare professionals and managers with little interest in telemedicine might have been less likely to respond [80]. However, we compared answers from the two waves of respondents, and there was no significant difference.

Another limitation is related to the fact that the questionnaire used mainly closed-ended questions as these are perceived as easier to complete and help to optimise completion rates [161]. This could have led to the omission of some factors due to the limited options available to respondents. However, we believe this is unlikely as the survey design was driven by an extensive literature review and discussed with the study team, which includes healthcare staff and academics with health service delivery and telemedicine expertise. Closed-ended questions may restrict the respondents to the choices provided. Still, we do not believe this is the case because we added several open-ended questions allowing participants to expand on their responses and greater freedom of expression [162].

Other limitation concerns the characteristics of the sample. Although the questionnaire was sent to different typologies of healthcare organisations, the 90% of the respondents belong to public health organisations. Moreover, the majority of respondents are from organizations located in Northern Italy, reflecting the uneven distribution of telemedicine services across the national territory [82]. Therefore, the results are not generalizable to the whole Italian health context. Also, the lack of comparison among different countries makes the findings of interest only to Italian healthcare management and policymakers.

The period in which the survey has been performed may also have caused biases. The strong conditioning in ensuring the social distance due to Covid may have emphasised positive perceptions towards telemedicine. A new survey in the current period could make the findings more robust. Finally, drivers, benefits, and challenges have been analysed only from the provider’s perspective, as patients were not included in the survey.

### Conclusions

In this study, we explore manager and healthcare professional perceptions on drivers, benefits, and challenges related to the use of telemedicine through a cross-sectional survey conducted in the Italian NHS during the Covid-19 pandemic. To our knowledge, this is the first study exploring healthcare professional and manager perspectives on the use of telemedicine at the national level over a range of different technologies and medical specialities.

Since the start of the Covid-19 pandemic, telemedicine is becoming an integral part of care delivery worldwide. It is vital to continue and improve telemedicine as a tool to supplement and augment in-person treatment and to ensure that both clinician and patient experiences are efficient, positive, and patient-centred. Several difficulties must yet be overcome before telemedicine may be considered a standard of care. To be successful, these initiatives necessitate guidelines and training, as well as careful consideration of technological hurdles and human interaction requirements.

### Electronic supplementary material

Below is the link to the electronic supplementary material.

Supplementary Material 1

## Data Availability

The questionnaire and the datasets used and analysed during the current study are available from the corresponding author upon reasonable request.

## Abbreviations

Covid-19: Coronavirus disease 2019
ED: Emergency Department
GDPR: General Data Protection Regulations
IN.GE.SAN.: Italian Association of Management Engineers in Healthcare
IRCCS: National Institutes for Scientific Research
NHS: National Health Service
