# Supplementary material for: Healthcare professional and manager perceptions on drivers, benefits, and challenges of telemedicine: results from a cross-sectional survey in the Italian NHS
Source: BMC Health Serv Res. 2023 Oct 18;23:1115. doi: 10.1186/s12913-023-10100-x (PMC10585875; doi:10.1186/s12913-023-10100-x)
Supplement: Supplementary file 1 — Supplementary Material 1 [file 12913_2023_10100_MOESM1_ESM.pdf]

Questionnarie on implementation of telemedicine services during the COVID-19 pandemic period

SECTION 1 – General Information on the organization and the telemedicine services implemented

1.1 What is your role in the organization?

1.2 Personal Information:

1.2.1 Sex
1.2.2 Age
1.2.3 Work experience [number of years]
1.2.4 Work experience in the current organization [number of years]

1.3 General Information on the organization:

1.3.1 Organization's location

a. Region

b. Province

1.3.2 Organization type

a. Hospital Enterprises (Azienda ospedaliera)

b. National Institutes for Scientific Research and Care (IRCCS)

c. Nursing homes

d. Local Health Authorities

e. Other [specify]

1.3.3 Organization ownership:

a. Private

b. Public

SECTION 2 – Drivers to the implementation of telemedicine services

2.1 Below is a list of enabling factors/drivers that have facilitated the introduction of new telemedicine services and/or the increase in the use of existing services during the pandemic. For each category (organization, technology, regulatory), please select the 4 most relevant drivers for you.

Organization:

- Containing the spread of the Covid-19 virus by reducing face-to-face contacts
- Availability of financial resources
- Organization's ability to plan and carry out the implementation phase, reorganize processes, and manage the change required by the introduction of technology.
- Ability to organize a multidisciplinary team to support implementation, defining roles and responsibilities.
- Availability of the technological infrastructure
- Introduction of pathways and/or protocols and usage guidelines.
- Previous experience with telemedicine
- Enhance the service level and the efficiency

Technology:

- Ease of use of the technology
- Efficacy and reliability of the technology
- Possibility to safely share information between stakeholders
- Compliance with current data security and privacy regulations
- Strong relationship with the technology supplier
- Availability of training programmes
- Possibility to customize the technology according to the patient's needs and condition.

- Cost of the technology

External context:

- Simplifications of regulations due to the Covid-19 pandemic
- Availability of external funds

2.2 Do you believe that telemedicine is a useful service even in the post-pandemic period?

a. Yes

b. No

Why?

2.3 What is your perception of the benefits brought by telemedicine during a crisis period, such as the pandemic?

Benefit	1 Significantly worsened	2 Worsened	3 No change	4 Improved	5 Significantly improved
Outpatient scheduling activities					
Efficiency of administrative processes					
Efficiency of care processes					
Quality of work (In terms of timing and organization)					
Waiting list					
Economic Impact					
Consultation time (Times required for the provision of health services)					
Availability and quality of patient records					
Collaboration with other healthcare professionals					

2.4 Below is a list of the main issues encountered by healthcare professionals when using the telemedicine service under consideration. Please provide a rating for the impact of these issues (the scale is ordered considering 1 as low impact and 5 as high impact).

	1	2	3	4	5
Technological obstacles					
Refuse to use telemedicine services					
Lack of trust in telemedicine technologies					
Internet connection not available					
Difficulty to use telemedicine technologies due to poor training					
Poor quality of telemedicine technologies					
Poor quality of Internet connection					

2.5 Patient acceptability and experience with telemedicine services

- Are patients open to the use of telemedicine services and have a good attitude toward using them?

Not keen at all	Not keen	Indifferent	Keen	Extremely keen

- Are patients satisfied of telemedicine services, after using them?

Very dissatisfied	Dissatisfied	Indifferent	Satisfied	Very satisfied

- Are telemedicine services offered by different healthcare providers in the area, enhancing patient choice?

Present in almost no healthcare provider	Present in a few healthcare providers	Present in a fair number of healthcare providers	Present in many healthcare providers	Present in almost all healthcare providers

2.6 Are there any barriers to using the service in question for certain patient groups? (e.g. neurological issues, language difficulties).

a. Yes

b. No

If yes, please specify what these barriers are.

The following table reports the literature that has ispired the items of the questionnarie

**SECTION 1 – General Information on the organization and the telemedicine services implemented**	Greenhalgh T, Wherton J, Papoutsi C, Lynch J, Court CA, Uk F, et al. Beyond Adoption : A New Framework for Theorizing and Evaluating Nonadoption , Abandonment , and Challenges to the Scale-Up , Spread , and Sustainability of Health and Care Technologies Corresponding Author : J Med Internet Res. 2017;19:e367.
**SECTION 2 – Drivers to the implementation of telemedicine services - Question 2.1**	Greenhalgh T, Wherton J, Papoutsi C, Lynch J, Court CA, Uk F, et al. Beyond Adoption : A New Framework for Theorizing and Evaluating Nonadoption , Abandonment , and Challenges to the Scale-Up , Spread , and Sustainability of Health and Care Technologies Corresponding Author : J Med Internet Res. 2017;19:e367.
**SECTION 2 – Drivers to the implementation of telemedicine services - Question 2.2**	Hollander JE, Carr BG. Virtually perfect? Telemedicine for COVID-19. N Engl J Med. 2020;382:1679–81.
**SECTION 2 – Drivers to the implementation of telemedicine services - Question 2.3**	- Driessen J, Castle NG, Handler SM. Perceived benefits, barriers, and drivers of telemedicine from the perspective of skilled nursing facility administrative staff stakeholders. J Appl Gerontol. 2018;37:110–20.<br>- Jacob C, Sanchez-Vazquez A, Ivory C. Social, organizational, and technological factors impacting clinicians' adoption of mobile health tools: systematic literature review. JMIR mHealth uHealth. 2020;8:e15935.
**SECTION 2 – Drivers to the implementation of telemedicine services - Question 2.4**	- Greenhalgh T, Wherton J, Papoutsi C, Lynch J, Court CA, Uk F, et al. Beyond Adoption : A New Framework for Theorizing and Evaluating Nonadoption , Abandonment , and Challenges to the Scale-Up , Spread , and Sustainability of Health and Care Technologies Corresponding Author : J Med Internet Res. 2017;19:e367.<br>- Keesara S, Jonas A, Schulman K. Covid-19 and health care's digital revolution. N Engl J Med. 2020;382:e82.<br>- Gilbert AW, Billany JCT, Adam R, Martin L, Tobin R, Bagdai S, et al. Rapid implementation of virtual clinics due to COVID-19: report and

	early evaluation of a quality improvement initiative. BMJ open Qual. 2020;9:e000985.
**SECTION 2 – Drivers to the implementation of telemedicine services - Question 2.5**	Bree E. H., Patients Perceptions of Telemedicine Visits Before and After the Coronavirus Disease 2019 Pandemics, 2021
**SECTION 2 – Drivers to the implementation of telemedicine services - Question 2.6**	He W., Zhang Z.J., Li W., Information Technology Solutions, Challenges, And Suggestions for Tackling The Covid-19 Pandemic International, 2021.
